# A Siamese Transformer Network for Zero-Shot Ancient Coin Classification

**DOI:** 10.3390/jimaging9060107

**Published:** 2023-05-25

**Authors:** Zhongliang Guo, Ognjen Arandjelović, David Reid, Yaxiong Lei, Jochen Büttner

**Affiliations:** 1 School of Computer Science, University of St Andrews, Scotland KY16 9AJ, UK; dgr20@st-andrews.ac.uk (D.R.); yl212@st-andrews.ac.uk (Y.L.); 2 Max Planck Institute for the History of Science, Boltzmannstraße 22, 14195 Berlin, Germany; buettner@mpiwg-berlin.mpg.de

**Keywords:** Siamese neural network, matching, deep learning, computer vision, machine learning, low-shot learning

## Abstract

Ancient numismatics, the study of ancient coins, has in recent years become an attractive domain for the application of computer vision and machine learning. Though rich in research problems, the predominant focus in this area to date has been on the task of attributing a coin from an image, that is of identifying its issue. This may be considered the cardinal problem in the field and it continues to challenge automatic methods. In the present paper, we address a number of limitations of previous work. Firstly, the existing methods approach the problem as a classification task. As such, they are unable to deal with classes with no or few exemplars (which would be most, given over 50,000 issues of Roman Imperial coins alone), and require retraining when exemplars of a new class become available. Hence, rather than seeking to learn a representation that distinguishes a *particular* class from all the others, herein we seek a representation that is *overall* best at distinguishing classes from one another, thus relinquishing the demand for exemplars of *any specific* class. This leads to our adoption of the paradigm of pairwise coin matching by issue, rather than the usual classification paradigm, and the specific solution we propose in the form of a Siamese neural network. Furthermore, while adopting deep learning, motivated by its successes in the field and its unchallenged superiority over classical computer vision approaches, we also seek to leverage the advantages that transformers have over the previously employed convolutional neural networks, and in particular their non-local attention mechanisms, which ought to be particularly useful in ancient coin analysis by associating semantically but not visually related distal elements of a coin’s design. Evaluated on a large data corpus of 14,820 images and 7605 issues, using transfer learning and only a small training set of 542 images of 24 issues, our Double Siamese ViT model is shown to surpass the state of the art by a large margin, achieving an overall accuracy of 81%. Moreover, our further investigation of the results shows that the majority of the method’s errors are unrelated to the intrinsic aspects of the algorithm itself, but are rather a consequence of unclean data, which is a problem that can be easily addressed in practice by simple pre-processing and quality checking.

## 1. Introduction

Among the many application domains in which the rapidly advancing fields of computer vision and machine learning have found their use is that of numismatics and ancient numismatics in particular. The term “numismatics” refers both to the academic study of coins, paper currency and tokens, as well as the hobby of collecting these items. Ancient numismatics concerns ancient coins in particular, that is, the coins of Ancient Greece, Rome, Celtic tribes, etc.

**Figure 1:**
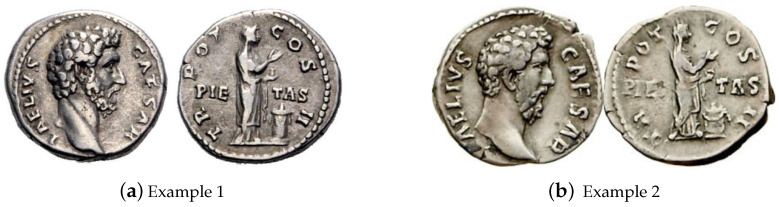
Examples of two different specimens of the same issue, namely of RIC 439 *Aelius* denarius.

Considering the inherently interdisciplinary focus of the present article, for the sake of clarity it is useful right at the start to introduce and define a few specialist terms from the vernacular of ancient numismatics, lest there be any confusion over their meaning due to their different use in everyday language. When referring to a “coin”, the reference is made to a specific and unique physical specimen. It is important to distinguish it from an “issue”, a more abstract notion that engenders all possible coins with the semantically identical design motifs. For example, two Roman Imperial coins may be said to correspond to the same issue if the same emperor, clothed in and oriented in a particular way, etc., is depicted on the obverse, and when the same inscriptions, and, say, deities in the identical poses and engaged in identical acts, etc., are shown on the reverse. Different issues of coins are uniquely referred by their identifiers from a variety of standard references, such as the Roman Imperial Coinage (RIC), as illustrated in Figure 1. A thorough summary of the relevant terminology for the non-specialist can be found in the recent review of computer vision challenges and problems in ancient numismatics by Arandjelović and Zachariou [1].

The determination of the issue that a particular coin corresponds to, that is its identification, is a task of foremost importance and the focus of the present article. In simple terms, it seeks to answer the question: “What coin is this?”. In most cases, this is a reasonably straightforward task for an expert, though there are exceptions, especially when the specimen in question is damaged and its issue is a rare one. For amateurs and especially beginner hobbyists, the challenge can be fiendish. For automatic, computer-based methods, the task has also proven to be a difficult one, both for reasons inherent in the problem as well as those that emerge from a variety of practical issues.

Given the aforementioned cardinality of coin identification, it is unsurprising that most of the work on the use of computer vision for ancient coin analysis has focused on solving precisely this problem. No more surprising is the overall approach that dominates the related literature. In particular, the structure of the coin identification problem is naturally seen through the lens of classification, with each issue seen as a class, thereby recasting the problem as that of assigning the correct class to an input image showing an unknown specimen [2,3,4,5]. Nevertheless, despite this superficial appeal of a classification-based approach to tackling the problem, it has become increasingly clear that its practical utility is highly limited by real world constraints pertaining to data availability. This becomes readily apparent as soon as the number of different coin issues is considered: there are over 50,000 for Roman Imperial coinage alone, and the number becomes far greater when Roman Republican, Roman Provincial, Ancient Greek, Celtic, etc., issues are included. It is practically impossible to obtain images of more than a small fraction of these, to say nothing of the need for multiple examples of each issue demanded by the present-day learning methods. There was some recognition of this major weakness of vision-based classification approaches already in the early years of work in this field [6], which has only become increasingly apparent since [7].

In the present paper, we propose a radically different approach whereby we learn to quantify the degree of belief that two specific specimens, e.g., a query and a gallery one, correspond to the same issue. Specifically, by learning what features should be extracted from an image of a coin for the purpose of its comparison with another coin and answering the question of whether they correspond to the same issue, in a manner independent of a specific comparison, we learn a fundamental representation of a coin that facilitates comparisons with new coins, that is coins that are not present in the training data set. By doing so, we learn a representation that does not rely on any *a priori* class structure. This means that if new images of coins are added to the reference gallery, be they additional examples of the already known classes or examples of an entirely new class, our model does not need to be retrained. It also means that our algorithm does not rely on multiple examples from the same class, as well as that its performance on underrepresented classes, which is a major issue for previous work, is not disadvantaged. The proposed approach provides a powerful way of making the most of the available information by facilitating different kinds of feedback to the user. Most obviously, if the best pairwise match is sufficiently high, the query coin can be attributed to the same issue as the corresponding match. On the other hand, if no such match is found, the gallery exemplars could be ordered by similarity, in a ranked retrieval fashion.

In terms of its technical underpinning, the present work pioneers two novelties. Firstly, this is the first work to describe the use of a Siamese architecture in this context. Secondly, as the baseline architectural component of each arm of the proposed Siamese network, we employ transformers, rather than convolutional neural networks that were featured in previous work [8,9,10].

## 2. Related Work

### 2.1. Automatic Ancient Coins Analysis Using Computer Vision and Machine Learning

Ancient coin analysis is a relatively new research domain for the application of computer vision and machine learning. The first forays into the territory were made a decade and a half ago by Zaharieva et al. [5]. The research effort in the field has since increased rapidly and dramatically [6,7,9,11,12,13,14,15,16], with an evermore varied range of specific tasks being targeted [1,6,17,18] and of modeling approaches [4,7,9,11,16,19].

Owing to the novelty of the problem, that is its unfamiliarity and the consequent lack of data, the earliest work centered its attention to what is arguably the simplest useful problem in ancient coin analysis which is *specimen classification* [5,20]. Thereafter, the focus in the field has quickly shifted towards the more challenging task *issue classification*. The reason for this lies in its broader practical interest as well as the greater technical challenge to automatic methods. Indeed, at present, the attention of nearly all existing computer vision work on ancient coin analysis is on issue classification [3,9,15,19,21], with a small number of notable exceptions [7,16,22].

In terms of technical methodology, the research on computer vision-based issue classification has largely mirrored the developments in computer vision more broadly. Thus, the initial attempts are addressing the challenge employed classical [2,4,5], that is non-learning, manually crafted features, e.g., SIFT [11] or wavelet transform [4] based descriptors, compared in a pairwise manner or aggregated using bagging [15]. Unlike in many natural image understanding applications, lacking in non-local geometric information, such representations quickly showed themselves to be insufficiently expressive for the task at hand. Hence, a number of follow-up methods sought to remedy this, for example by crafting geometric context aware features [19,21] or by aggregating local features in a spatially sensitive manner [15,23]. While effecting an improvement, such attempts have still proven insufficiently effective in producing a viable real-world solution; neither type of approach achieved sufficient expressive power nor robustness to the common challenges present in the data [23], with the latter kind of algorithms also suffering from sensitivity to the precise orientation of the coin, its centering, and variations across dies of the same issue [18].

Reflecting the trends in computer vision more broadly, a major leap in performance came with the adoption of deep learning [8,24]. Since then, a series of authors have demonstrated the power of deep learning, convolutional neural networks (CNNs) in particular, to address the key challenges that were thitherto insurmountable by classical computer vision approaches, namely intra-class variability caused by damage, the minting process, and different dies; and illumination and other photometric changes [3]. Complementing the CNN-based work, Zachariou et al. [16] recently demonstrated how a generative adversarial network can be used to synthetically reconstruct images of undamaged coins from original images of damaged specimens, thereby further directly addressing the major challenge that wear poses to automatic methods [23].

Notwithstanding the noted methodological improvements in the technical aspects of the methods proposed as a means of addressing the problem of ancient coin attribution, as recently pointed out by Cooper and Arandjelović [7], what has remained all but unchallenged in the 15 years of work is the fundamental manner of approach to the problem. In particular, the published work thus far frames attribution as a classification problem: given a known set of classes, each with images of exemplars, the correct class of an image of a novel specimen is sought. This is a reasonable framing if the classification is very coarse, e.g., by the denomination of a coin [22], when the premises of the setting are easily satisfied. Arguably, the strategy can also be defended when the classification is semi-coarse, e.g., when a class corresponds to the issuing authority on the coins’ obverses [8]; examples of coin images of only the rarest of issuers, which are few in number, may be problematic. However, when fine attribution is of interest, which is something that numismatists, be they hobbyists or professionals, are interested in first before any further analysis is conducted, then it can be readily seen that the classification paradigm is no longer viable. The reason lies in the very large number of emergent classes and the difficulty—or rather, impossibility in practice—of obtaining exemplars of but a small fraction of their total number. As noted by Arandjelović and Zachariou [1], Online Coins of the Roman Empire (OCRE; see http://numismatics.org/ocre/ accessed on 1 May 2023), a joint project of the American Numismatic Society and the Institute for the Study of the Ancient World at New York University, lists 43,000 published issues, and the true count is likely to be even greater. The only work to date that has tackled this challenge directly is that of Cooper and Arandjelović [7]. More precisely, what Cooper and Arandjelović propose is the first step towards overcoming the aforementioned problem, introducing a text mining and CNN-based method to learn to recognize the semantics of different elements depicted on coins, thereby transferring the representation from the image domain to the text one, the latter being far more abundant in data, easier to interpret, and simpler to match or otherwise analyze. Although their approach has demonstrated promising performance on a small number of frequently encountered concepts, at present there still remains a large gap between the method’s currently demonstrated capabilities and those needed to make the technology practically useful for the task of exact issue identification.

The method we introduce in the present paper emerges from the nexus of the described weaknesses of the previous work, while also drawing strength from ideas that have previous been showed to yield promising results. In particular, in order to overcome the difficulties associated with an extremely large number of classes (that is, coin issues), instead of seeking to learn a representation that distinguishes a *particular* class from all the others (classification), herein we seek a representation that is *overall* best at distinguishing classes one from another, thus relinquishing the demand for exemplars of *any specific* class. This leads to our adoption of the paradigm of pairwise coin matching by issue, rather than the usual classification paradigm, and the specific solution in the form of a Siamese neural network [25]. Furthermore, while adopting deep learning, motivated by its successes in the field and its unchallenged superiority over classical computer vision approaches, we also seek to leverage the advantages which transformers have over the previously employed convolutional neural networks [26], and in particular their non-local attention mechanisms, which ought to be particularly useful in ancient coin analysis by associating semantically but not visually related distal elements of a coin’s design (i.e., in the legend and in the pictorial motif).

### 2.2. Siamese Neural Networks

**Figure 2:**
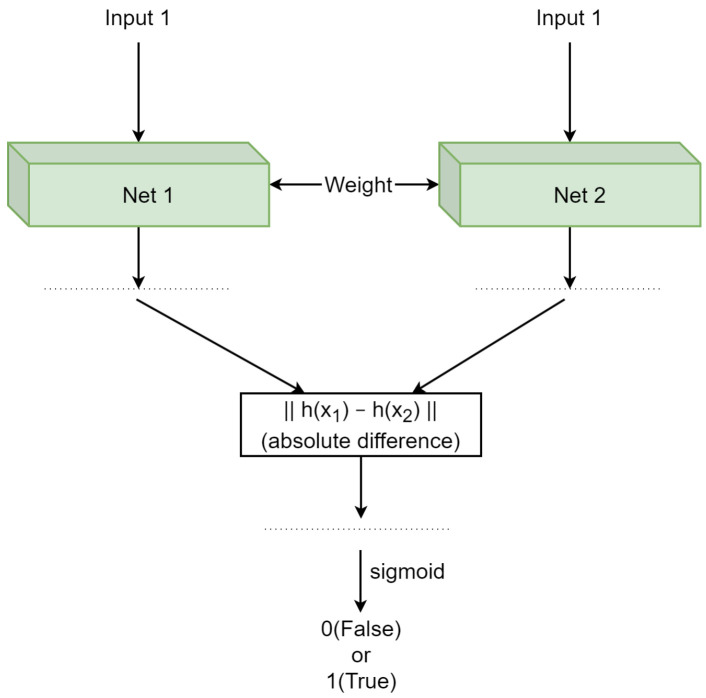
The architecture of a Siamese neural network comprises two mutually mirroring processing streams consisting of two identical neural networks with shared hyperparameters [25].

A Siamese neural network (SNN) [25], illustrated in Figure 2, is a kind of a coupling architecture. Comprising two mutually mirroring processing streams, it is based on two identical neural networks with shared hyperparameters. When fed two inputs from the same input space (images of coins in our case), it learns to produce their discriminative representations in a high-dimensional space. A comparison of these representations is also learned, ultimately producing a similarity score between them and thereby of the two original inputs they correspond to as well [25,27].

Siamese neural networks were first proposed in the context of signature verification, which is for the determination of signature forgeries [25]. Subsequently, they have been adopted and proven successful in a wide variety of other matching-based tasks, such as gait recognition [28], sporting activity recognition [29], natural language processing [30], object reconstruction [31] and others. Appropriately applied, Siamese neural-network-based algorithms have been shown to improve classification accuracy and enhance rejection quality compared with traditional convolutional neural networks [32]. Moreover, SNNs can significantly reduce the number of hyperparameters during model training and improve operational speed while maintaining their superior accuracy performance [33].

### 2.3. Transformers

The transformer [26] is a deep learning architecture originally proposed for natural language processing (NLP) applications, which revolutionized the field and led to new state-of-the-art models while also reducing training times for large data sets. Google’s Bidirectional Encoder Representations from Transformers (BERT) model [34] has been used to improve the search functionality for more complex queries. OpenAI’s Generative Pre-trained Transformer 3 (GPT-3) [35] became the largest neural network ever constructed, making headlines with its impressive ability to generate text that appeared to have been written by humans.

The transformer follows a similar encoder–decoder architecture to previous models, in which one sequence of tokens, representing words in a sentence, is used to generate another sequence (e.g., a translation of the sentence). What is special about the transformer architecture is that, unlike its predecessors, it does not use convolutional layers or recurrent connections, but instead largely relies on self-attention [26], a mechanism for focusing on information relevant to the current task. An attention unit’s role is to map equal-length sequences of query, key and value vectors to a sequence of context vectors, each of which is a weighted mean of the value vectors, weighted towards those that are most relevant to the corresponding position in the sequence for the given task. Attention weights are computed using three matrices that are learned during training, $Q\in R^{n\times d_{k}}$ , $K\in R^{n\times d_{k}}$ , and $K\in R^{n\times d_{v}}$ [26], with their rows, respectively, being query, key, and value vectors, where *n* is the maximum sequence length, $d_{k}$ is the dimensionality of the query and key vectors, and $d_{v}$ is the dimensionality of the value vectors. In a translation context, the query vectors correspond to words in the target language, whereas the key and value vectors would correspond to words in the source language. Let the words of the input sentence be represented by the rows of $X\in R^{n\times d}$ then the learnable embeddings $W^{K}\in R^{n\times d_{k}}$ and $W^{V}\in R^{n\times d_{v}}$ project the input X to the key matrix $K=XW^{K}$ and the value matrix $V=XW^{V}$ [26]. Let the words of the translated output up to the current token be represented by $Y\in R^{n\times d}$ , then a learnable embedding $W^{Q}\in R^{d\times d_{k}}$ projects Y to the query matrix $Q=YW^{Q}$ [26].

The transformer architecture uses scaled dot-product attention whereby how much the *j*-th value vector contributes to the *i*-th context vector is determined by the dot-product of the corresponding query and key vectors. The dot-products are scaled by $1/\sqrt{d_{k}}$ [26], lest they become too large, resulting in problematically small gradients. Softmax is applied to the dot products to obtain positive weights that sum to one. The attention weights are then multiplied by the value vectors to obtain the context vectors.

Previous language models that used recurrent neural networks (RNNs) struggled to learn long-range dependencies between words in long sequences, because it processed tokens sequentially, meaning that any state passed forward in the network had to encode the entire sequence up to the current token, which became less effective the longer the sequence was. In the transformer model, the self-attention mechanism operates over the entire sequence of input symbols, so it is equally able to handle dependencies over any range. Another issue with sequential processing is that training could not be parallelized as effectively. The transformer employs multi-head self-attention (MSA), running multiple identical, but separately parameterized, self-attention units (“heads”) in parallel. This allows it to attend to different regions for different representations concurrently, which would not be possible with a single head, as the weighted mean of many many points of interest would result in a lack of focus on anything in particular. The output of a MSA unit is the concatenation of the output vectors from the individual heads, projected by a matrix back to vectors of dimensionality $d_{model}$ , which is constant throughout the transformer.

The encoder in the transformer consists of *N* identical layers. The input to the decoder is an embedding of the input sequence and the output feeds into the decoder. Each layer in the encoder has two sub-layers: the first is an MSA unit; the second is a fully connected feed-forward network (FFN), consisting of two linear transformations with a ReLU activation in between. Skip connections around each sub-layer are used. These are a widely used feature in deep learning that help with vanishing gradients. Furthermore, skip connections have been shown to improve the ability to learn by flattening the loss landscape [36]. As well as skip connections, layer normalization is applied to each sub-layer, as this has been show to improve training times [37].

The decoder also consists of *N* identical layers with further sub-layers. As for the encoder, a MSA unit and a FFN are two of the sub-layers, and skip connections and layer normalization are used. The extra sub-layer is a multi-head attention (*not* self-attention) unit, for which the key and value matrices come from the output of the encoder and the query matrix comes from the output of the preceding MSA unit. This is how information flows from the encoder into the decoder. The input to the decoder comprises the tokens of the output sequence.

Unlike the structure of an RNN or a CNN, the transformer architecture does not implicitly contain any notion of position for the input data. Instead, positional encodings for each token are added to the input embeddings that are passed into the encoder. Whereas an RNN or a CNN has a strong inductive bias towards locality, a transformer has few inductive biases, so it must learn the significance of positional relationships during training. This lack of a strong inductive bias makes transformers rather generic and able to model long-range dependencies, at a cost of worse performance for small training sets for which sensible inductive biases can be beneficial. Since transformers need a large amount of training data, transfer learning is typically used: a model that has been pre-trained on a large but more generic data set is fine-tuned with training data for a specific task, enabling it to make use of previously learned generalizations and thus avoiding the need for task-specific training from scratch.

#### Vision Transformer

The vision transformer (ViT) architecture [38] is a direct descendant of the transformer architecture. It follows the original transformer architecture closely, enabling existing efficient transformer implementations to be used with ease. Whereas transformer was designed for sequence-to-sequence language tasks and therefore had an encoder and a decoder, ViT is used for image classification tasks and so it only has an encoder, to which tokens representing an image are provided as input. One of the main design decisions for ViT was how to embed the image. A naive implementation of self-attention would allow each pixel to attend to every other pixel, resulting in $O(n^{2})$ time and space complexity for images of *n* pixels. This would be prohibitively expensive, so a simplification has to be made. In the case of ViT, the simplification is to use image patch embeddings as the input tokens rather than pixels. Each image of width *W* and height *H* is divided into patches of P×P pixels, resulting in $N=WH/P^{2}$ patches, which is a small enough number to make self-attention across patches feasible. The square patches are flattened to vectors, which are projected by a learnable embedding to vectors of dimensionality $d_{model}$ , which is the size of the vectors used throughout the layers of the encoder.

As ViT is used for classification, an additional learnable class token embedding is passed in to the encoder as the zeroth “patch embedding”. After the final layer of the encoder, an additional FFN is added, which maps the context vectors from the zeroth position in the last layer to the image classes. During training, the network learns to encode in these output context vectors a representation of the image that is then used for classification purposes. During fine-tuning, a different FFN is used to project this image representation to the classes specific to the problem domain. In the FFNs of the encoder, GELU is used as the activation function, whereas the Transformer uses ReLU, but the authors offered no explanation for this modification. As for the Transformer, ViT includes positional information in the data passed to the encoder, otherwise spatial relationships between patches could not be learned. A 2D-aware positional embedding offered no significant improvement over a 1D positional embedding, so a 1D positional embedding is used instead, leaving it to the network to learn how the patch positions were spatially related to one another.

ViT was compared with CNNs [38], specifically ResNets [39], and hybrids of ViT and CNNs, for which the input sequence to the ViT was formed from the feature maps of a trained CNN, rather than image patches. While the hybrids outperformed ViT for smaller data sets, (presumably because the features already encoded at least local structure within the data), this performance difference vanished for larger data sets, demonstrating the ability of ViT to learn complex features without a strong inductive bias towards local features. As the size of the training dataset was scaled up to 300 million images, the performance of ViT continued to increase without reaching saturation, showing that more data are better when it comes to training ViT models.

For CNNs, the size of dependencies that can be represented by a feature at a given layer is limited by the receptive field for the feature. The size of the receptive field increases with depth. In contrast, ViTs can model long-range dependencies in their lowest layers. By visualizing the mean distance in the image space over which information was integrated for a given layer of ViT, it was found that some heads even in the lowest layers of ViT modeled long-range dependencies, whereas others were highly localized [15]. For the hybrid models tested, highly localized attention was less pronounced, suggesting that the role played by the highly localized attention heads was similar to that played by early convolutional layers in a CNN.

## 3. Proposed Methodology

To contextualize the design choices introduced in this section, remember the key practical problems that the present work seeks to address. The foremost one of these is the challenge of the input belonging to a class (coin issue) that was not present during training, which is something that no existing work has recognized fully or attempted to tackle. The second challenge is that of dramatic class imbalance, which has been noted in the relevant literature [7], but which has been left wanting in terms of a practicable and effective solution. The models we introduce here, all based on a Siamese architecture underlain by visual transformers, address both of the aforementioned challenges in a principled manner.

### 3.1. Proposed Network Architectures

In the present work, we propose and compare two different SNN-ViT-based architectures for ancient coin matching. The first one, hereafter referred to as the Single Siamese ViT, performs matching of obverses and reverses independently. The second architecture, hereafter referred to as the Double Siamese ViT, compares both the obverses and reverses, and integrates the obtained side-based scores into a single coin based similarity. In both cases, we employ a base ViT model pre-trained on imagenet-1k.

#### 3.1.1. Single Siamese ViT

**Figure 3:**
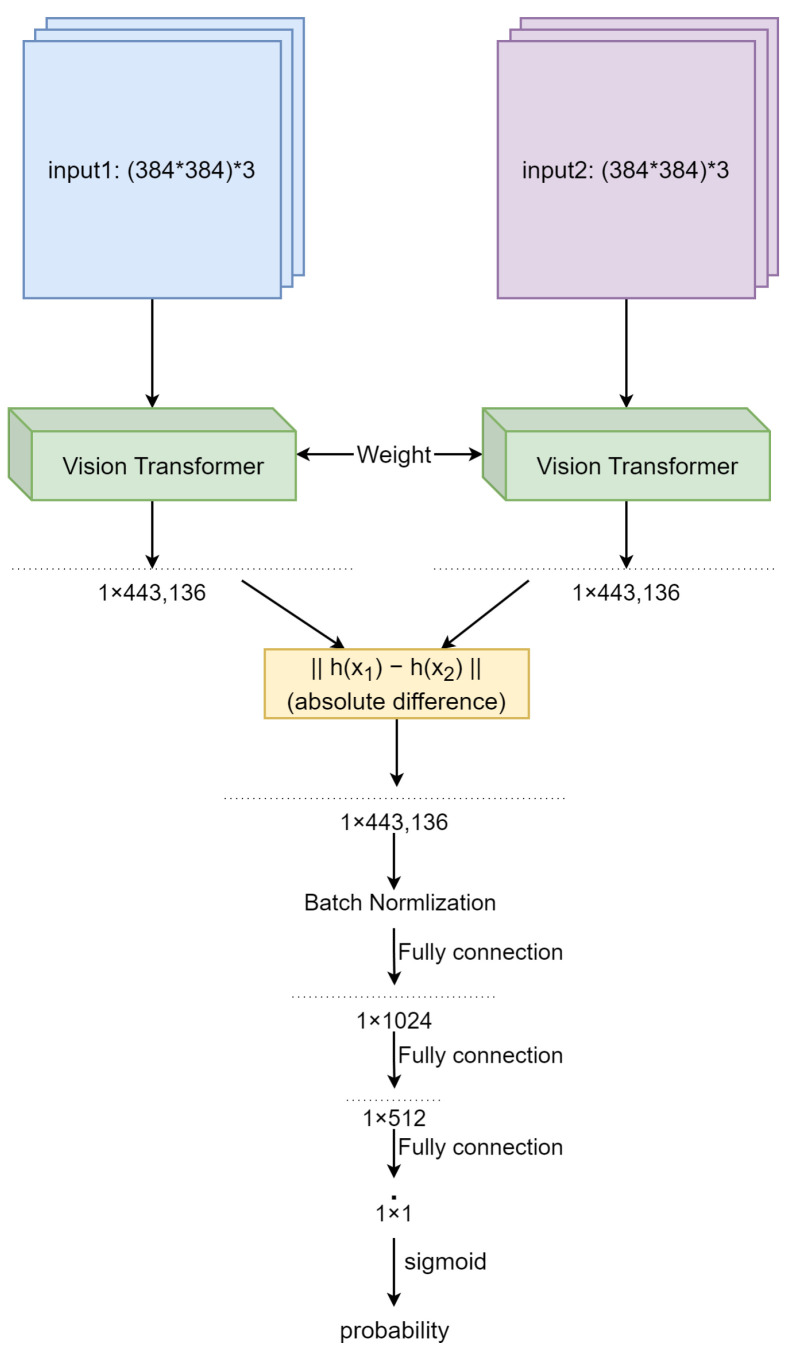
The architecture of Single Siamese ViT.

For the architecture of our obverse of reverse matching Single Siamese ViT, we adopted and adapted a generic Siamese Network as follows. Firstly, the backbone network of the network was replaced with a pre-trained ViT. Next, the semantic layer outputs of the two ViT models comprising the network and corresponding to the two streams processing the two inputs (obverses or reverses being matched) were flattened, and the absolute distance between them computed. Then, three linear layers and one batch normalization layer were used to reduce the dimension and produce the provisional output. Lastly, this output was passed through the sigmoid function to obtain a quasi-probability match measure, i.e., a number between 0 and 1. The architecture of this network can be seen in Figure 3.

#### 3.1.2. Double Siamese ViT

**Figure 4:**
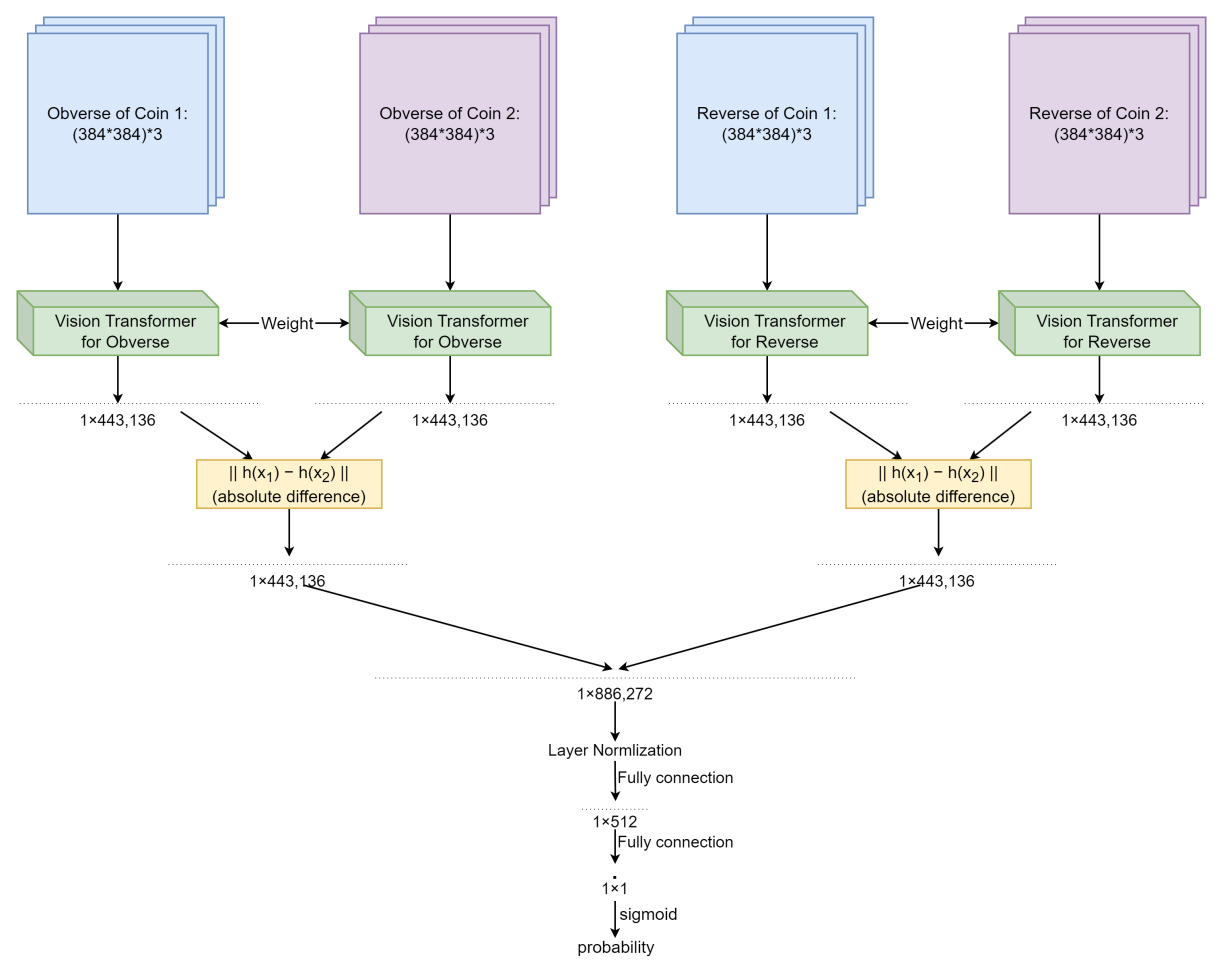
The architecture of Double Siamese ViT.

Our Double Siamese ViT, which processes both the obverses and the reverses of two coins that are matched, is for the most part based on the already described Single Siamese ViT model, with changes and additions to the final layers of the network. The two Single Siamese ViT networks remain identical up to and including the computation of the absolute difference of their semantic layers. Following this stage, their outputs are concatenated and layer normalization applied to the concatenated result. This is followed by two fully connected layers. As before, the provisional output of the second layer is passed through the sigmoid function, thereby obtaining a quasi-probability matching score on the coin level. The architecture of this network can be seen in Figure 4.

### 3.2. Training Methodology and the Organization of Training Data

No less important than the architectures of the learning models to the success of our overall approach is the manner in which the training of these models is performed, that is, the methodology employed to make the best use of the available training data. In the present work, we had a total of 20,000 training images of coins (specimens) available, spanning 7000 different issues. With the conventional, classification approach pursued by the previous work, this would lead to 7000 classes. On the one hand, this is an imposing number of classes. Yet, it is vastly smaller than the number of potential classes, that is, the different Roman Imperial coin issues. What is more, there would have been a major challenge posed by high imbalance and few exemplars even for a large proportion of issues included.

**Figure 5:**
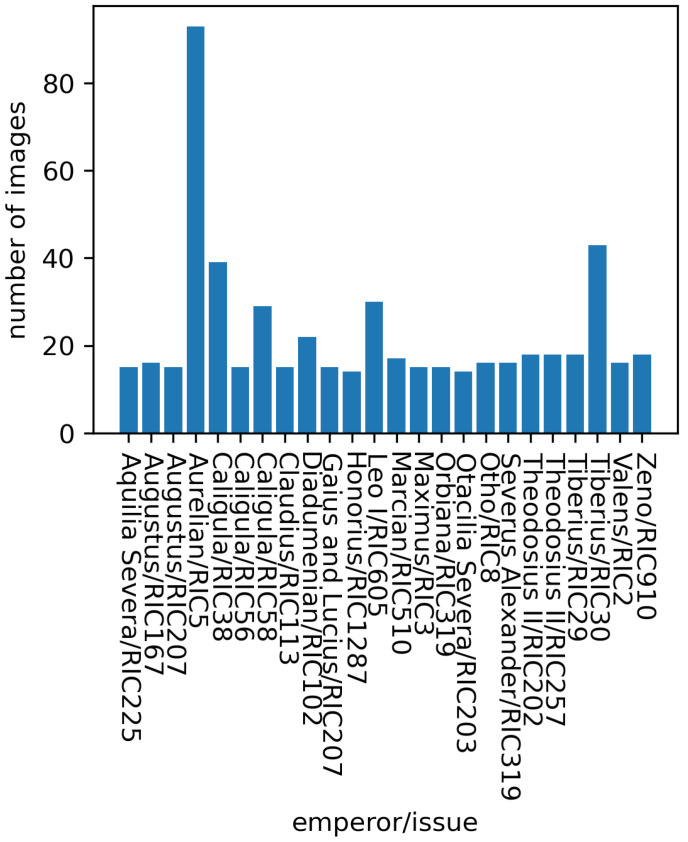
The data distribution of the train set.

In contrast is the approach we advocate herein, whereby the machine learning model learns coin characteristics, which allows for the discrimination of same issues vs. different issues on a pairwise basis. The challenge of class imbalance is inherently avoided (with a caveat upon we will elaborate shortly), as is that of a large number of classes. However, a new practical choice emerges, that of designing the training process in a manner that is feasible. In particular, the space of possible training inputs (coin pairs) is enormous, totaling $C_{20000}^{2}$ = 199,990,000 combinations. Even if only a single sample of each coin issue is considered, there are over $C_{7000}^{2}$ = 24,496,500 combinations, which is clearly impractical. However, the inherent non-reliance of our approach on the presence of any specific issue allowed us a straightforward way of dramatically downsizing the actual training set. In particular, for training we considered only those issues containing over 20 samples. Out of these, we randomly chose 14 for training our model, 3 for validating it, and the remaining 3 for its final testing, these being entirely unseen during the training-validation process. Doing this resulted in a training set containing 542 images representing 24 issues, and the test data set for the evaluation of the final model consisting of a total of 7605 issues over 196 individuals (emperors, empresses, etc.) depicted on their obverses. Figure 5 shows the distribution of the training set.

**Figure 6:**
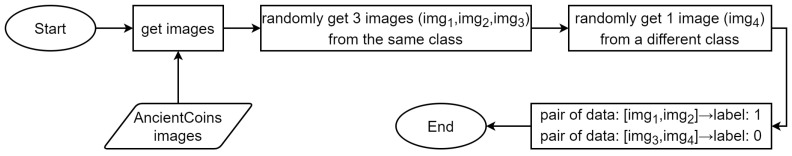
The flow chart for organizing data set.

The process just described adequately addresses the challenges of a large number of classes, the consequent need for a vast amount of training data, and, partially, that of class imbalance. The latter challenge is at this stage overcome only partially because it is still the case that the number of all same-issue pairs still outnumbers the number of all different-issue pairs, risking the over-weighting of correct decisions when the input coins do belong to the same issue relative to the decisions when they do not. However, considering that the exemplar count of both is large, this remnant imbalance is resolved rather effortlessly. In particular, all that needs to be done and indeed what we did in this work, was to perform balanced sampling of same-issue and different-issue pairs. The flow chart of this process is summarized diagrammatically in Figure 6.

## 4. Results and Evaluation

Having described the technical specifics of our models in the previous section, we now turn to the empirical evaluation of the same. We start by presenting the results obtained using our Single Siamese ViT, separately matching coin obverses and reverses, and then follow up with an assessment of our Double Siamese ViT, which matches coins holistically, that is, both obverses and reverses jointly, thereby de facto matching the corresponding issues themselves.

### 4.1. Data

In this work, we made use of the large data set of ancient coin images provided by the Ancient Coins Search Engine (https://www.acsearch.info/ accessed on 1 May 2023) for research purposes, which has been used in a number of previous research efforts [10,16]. This corpus consists of high-quality images obtained in rather controlled environments, usually with a uniform background, favorable lighting, natural coin alignment, etc. Whilst including a variety of non-Roman coins (Greek, Celtic, and Byzantine, among others), as well as Roman non-Imperial ones (namely Provincial and Republican), the Roman Imperial coins included span the entire time period of the Empire and cover most of the obverse figures depicted on them and listed in the Appendix A in Table A1.

The acsearch data set in its raw form comprises images with the associated free-form textual descriptions as provided by auction houses. In other words, there is no semantically organized meta-information that would allow us to identify the entries that are of interest herein, namely Roman Imperial coins with the corresponding RIC identifiers. Considering the large size of the corpus and hence the impracticality of this being done by a human, we achieved the desired extraction automatically. In the processing of a single candidate entry, we first searched for the presence of the names listed in Table A3 in the associated text file. If none were found, or there were multiple different names found, the entry was not included in our experiments. The absence of a find suggests a coin other than Roman Imperial, whereas multiple matches meant that the entry was not a single item but a coin lot, or simply that there was ambiguity, which would have required a much more semantically nuanced data extraction method than was necessary for the extraction of a sufficient number of entries for the purposes of the present work. For entries that contained a single matching name, we next searched the text file for the RIC identifier using the regular expression “RIC.*?∖d”. Any entries without a match were also discarded; this would happen when another standard reference other than RIC was used (e.g., Roman Silver Coins (RSC)), or when a non-standard format for RIC was used. Finally, the images of the qualifying entries were split into two images, the obverse and the reverse, by dividing the image horizontally half way. No further efforts to register the resulting images were made, leaving any variation due to translation to be learned by our transformer-based, and hence patch-ordering-independent, model.

### 4.2. Single Siamese ViT

Recall that the proposed Single Siamese ViT is designed to match only a single side of a pair of coins, that is, either their obverses or reverses, and is accordingly trained with the corresponding sides only. Understanding the performance of this network, considering that it forms the basis of our more complex model, the Double Siamese ViT, evaluated subsequently, is crucial for understanding and contextualizing the performance of the latter. Further to providing insight into the power of the architecture itself and the manner in which we approach training, the findings presented here are also key to understanding how the network deals with the challenges presented by obverse and reverse motifs, which differ substantially. In particular, while obverses almost without an exception depict the head or the bust of a person (emperor, empress, heir, etc.) surrounded by a circularly arranged legend (text), the range of motifs on reverses is far more varied and complex, showing scenes (e.g., funeral pyres, bridges and building, rivers and forests, deities, etc.).

#### 4.2.1. Obverse Matching

**Figure 7:**
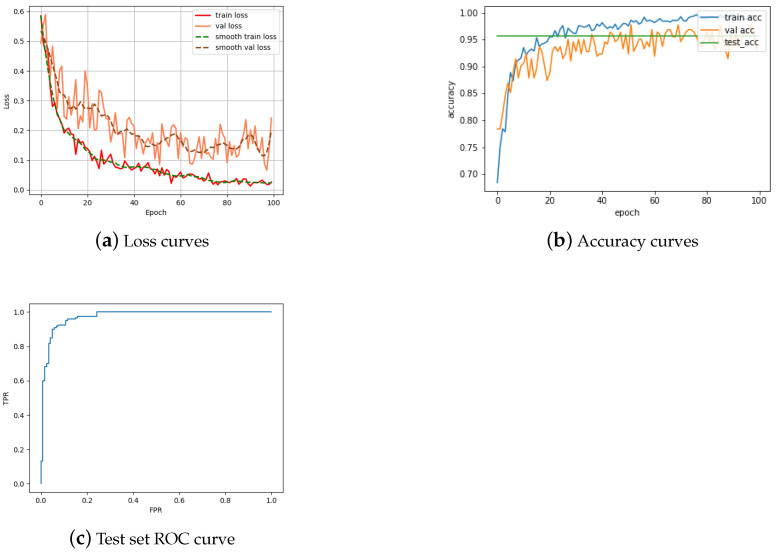
Performance characteristics of the proposed Single Siamese ViT on the obverse matching task.

We turn our attention to the task of obverse matching first. As the plot in Figure 7a shows, save for stochastic oscillations, we observed a decrease in the training loss throughout the training process, that is, with additional training epochs. Nevertheless, the rate of loss decrease slows down significantly by epoch 100, which gives reassurance that not much further benefit would be conferred by longer training. The concurrent and mirroring behavior of the validation loss indicates successful learning and a well-fitted ultimate model. Indeed, evaluated on the test set, the model achieves the accuracy of 95.73%, which matches that of the final validation accuracy and is expectedly somewhat lower than the final training accuracy (see the accompanying plots in Figure 7b); the impressive corresponding ROC curve is shown in Figure 7c. Our test set accuracy significantly exceeds that achieved by previous work on the obverse matching task, e.g., that reported by a CNN-based approach of Schlag and Arandjelović [8]. Still, our result is even more astounding given that the exact problem addressed by Schlag and Arandjelović is weaker than ours: whereas they merely seek to match the depicted obverse persons’ identities, we tackle the more specific matching of the precise obverse issues, which requires not just the matching of the corresponding persons’ identities, but also of their dress and adornments, as well as obverse legends.

#### 4.2.2. Reverse Matching

**Figure 8:**
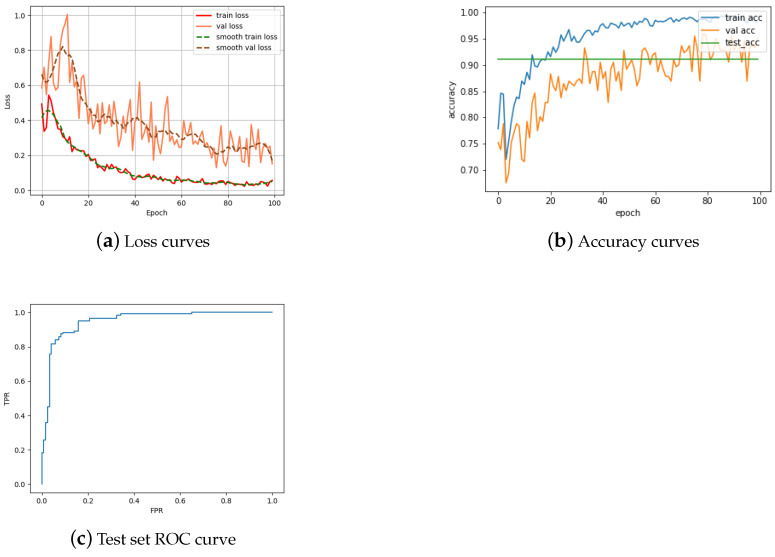
Performance characteristics of the proposed Single Siamese ViT on the reverse matching task.

We next turn our attention to the task of reverse matching. As in the previously described experiments on coin obverses, in training we observe a declining loss, both on training and validation data, throughout the training process, with the decline slowing down considerably by the epoch 100; see Figure 8a. However, the differences between the two training processes are noteworthy and highlight a few insightful points, which we expected from the theory as explained previously. Firstly, notice that model improvement slows down earlier in the case of reverses, suggesting an inherent limitation in the model to learn further semantic nuance. This is important when one also observes that the final model loss, both on training and on validation data, ends up being significantly higher in the case of reverse matching than obverse matching, offering substantiation to our expectation that the greater complexity of reverse motifs is inherently more difficult to learn. These interpretations are additionally corroborated by the accuracy plots shown in Figure 8b. In particular, while the reverse training accuracy is almost insignificantly lower than the obverse training accuracy, the equivalent discrepancy between the validation accuracies is somewhat larger (while still small), and the final accuracy on the test data set even more so. The final accuracy achieved is 91.03% (compare this with 95.73% for obverses). The corresponding ROC curve is still impressive, though also not quite as close to the ideal as that achieved on the obverse matching task.

### 4.3. Double Siamese ViT

**Figure 9:**
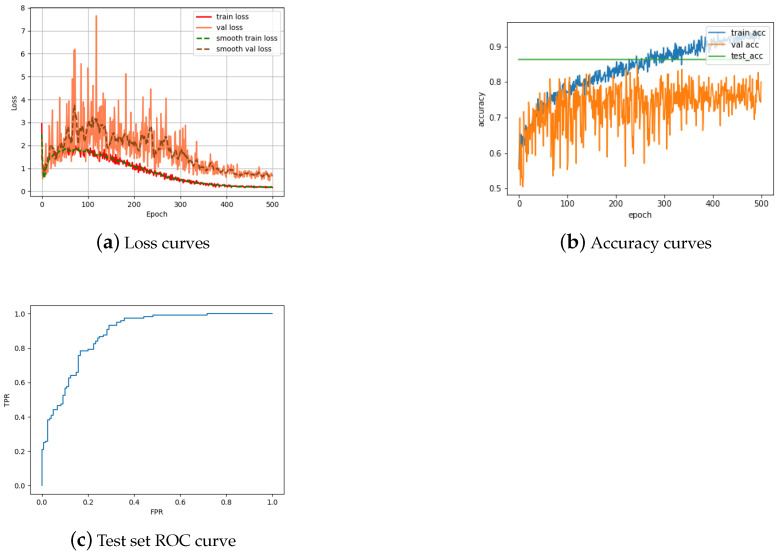
Training behavior of our Double Siamese ViT.

Empowered with an understanding of the strengths and weaknesses of our Single Siamese ViT, we finally evaluate the main model introduced in the present paper, namely our Double Siamese ViT, which uses Single Siamese ViT networks as its core building blocks. To overcome the computational challenge of training such a large network from scratch, and the problems associated with issues such as those of vanishing gradients and overfitting, we adopt the trained Single Siamese ViT networks of the previous section (one for the matching of images of obverses and one for the matching of images of reverses), freeze their weights, and train only the remainder of the architecture. Owing to this training design choice, we now observed rather different behavior of losses during training, as shown in Figure 9a. In particular, unlike during the training of the Single Siamese ViT on obverses and reverses, respectively, in Figure 7a and Figure 8a, here we note an initial increase in losses, which start to decline only following a peaking around the epoch 100. Thereafter, the behavior becomes much more familiar, the losses steadily declining following the peak, and settling by the epoch 500 (note the five-fold greater number of epochs needed as compared to the Single Siamese ViT). The greater challenge addressed by the Double Siamese ViT is also apparent from the accuracy plots in Figure 9b, with the training accuracy steadily and rather rapidly improving throughout the training process, reaching close to 100% performance by the epoch 500, contrasting the lack of validation accuracy improvement from as early as the epoch 100. The accuracy of the final, trained model was found to be 86.36%, which is impressive and far greater than that achieved by previous work on much simpler tasks, though understandably lower than the accuracy of the Single Siamese ViT on either of the sub-tasks of obverse or reverse only matching. Similar observations apply to the obtained ROC curve shown in Figure 9c.

#### Further Model Probing

While our Double Siamese ViT model achieved outstanding results, vastly outperforming the existing state-of-the-art, it expectedly did not perform perfectly, i.e., it could not match a human expert on the task of coin issue matching. Hence, we sought to understand the model’s performance with more nuance and gain insight into its strengths and weaknesses, both being important for future work and any potential improvements to it. As the first step towards this goal, we performed an additional set of experiments. In these experiments, we sought to match issues using (i) obverses only and (ii) reverses only, using our Single Siamese ViT model, and compared the results on an emperor-by-emperor basis with the joint matching performed by the proposed Double Siamese ViT model. Note that the single-side-based matching done here was different than that described in the previous section. In particular, while in the previous section we also used the Single Siamese ViT model to perform single side matching (obverse or reverse), a match was considered correct if it matched that side correctly. In contrast, here we take the match to extend to the entire issue. Clearly, in general, the information from only one side of the coin is insufficient to fully specify an issue, though in some cases it is (some issues feature obverse or reverse motifs or details not found on other issues), which is why a human would always examine the coin in its entirety when performing attribution. That is precisely the value of the approach taken in this experiment. Specifically, by making an emperor-by-emperor comparison, our findings illuminate both the magnitude of the value added of a joint consideration of both coin sides, as well as give insight into when this is most helpful. For example, we expected that the greatest gain would be seen when an issuing authority on the obverse is featured on many different issues, as well as when a particular motif recurs over long stretches of time (this would be the case, for example, for generic propaganda about prosperity and the virtues of the Empire, but not with one-of-a-kind events such as military victories).

**Figure 10:**
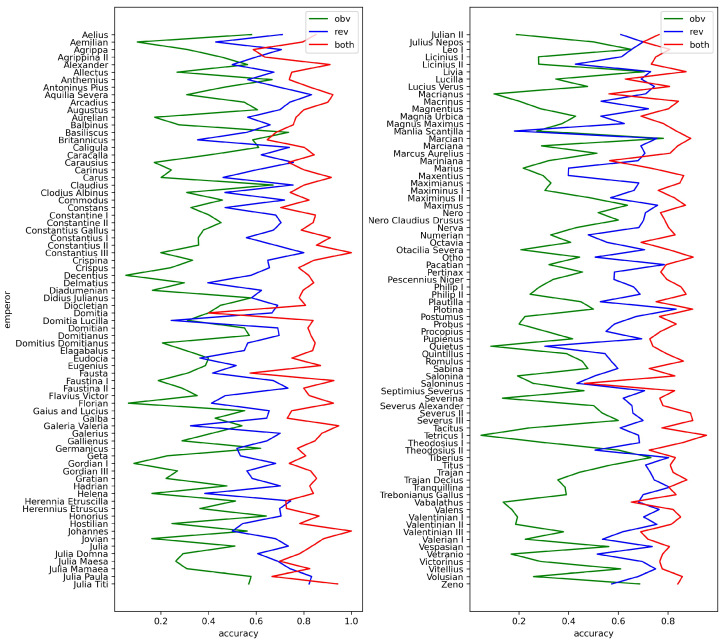
Summary of matching accuracy shown averaged over each issuing authority shown on the obverse.

The full numerical results over matching accuracies averaged across issuing authorities are presented in Table A2, Table A3 and Table A4; a graphical summary is shown in Figure 10. The immediately apparent finding is, as hypothesized, that the Double Siamese ViT model, i.e., issue matching using both coin sides, significantly outperforms both Single Siamese ViT models, i.e., issue matching using either side in isolation. The improvement is observed both on average as well as in the case of nearly every issuing authority; we shall return to the the unusual exceptions shortly. Observe that even when both single-side-based predictions perform poorly, their complementary role in the unique determination of an issue is reflected in the virtually universally highly accurate prediction when a coin is handled in a holistic manner. Indeed, the advantage of the Dual Siamese ViT model is particularly apparent when at least one of the two single side predictions is poor, e.g., because there are numerous issues under the same issuing authority (demonstrated by the poor predictive performance of obverses) or when a reverse motif is repeated across many issuing authorities (demonstrated by the poor predictive performance of reverses).

### 4.4. Analysis of Problematic Issuing Authorities

We noted previously that while the issuing authority averaged matching performance of the Dual Siamese ViT model is nearly universally high, there are some exceptions. In order to gain insight into this finding and discover a potential weakness of the proposed method, we identified the 15 most problematic issuing authorities, judged by the lowest average matching scores as per Table A4 and Figure 10, and manually examined the corresponding coin images. We readily identified a number of reasons for the aforementioned poorer than expected performance, most of which have to do with the quality of the available data rather than with any inherent, technical aspect of the proposed model itself. This is further elaborated next.

#### 4.4.1. Physically Incomplete Specimens

**Figure 11:**
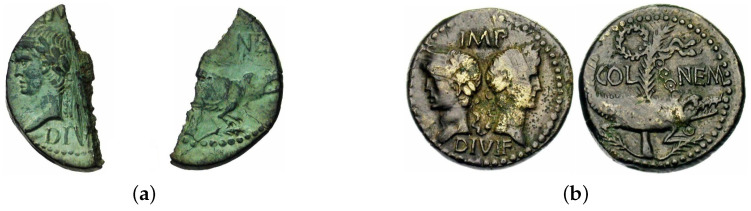
Examples of RIC 158 of *Augustus*. (**a**) An incomplete specimen of RIC 158 of *Augustus*. (**b**) A good condition specimen of RIC 158 of *Augustus*.

**Figure 12:**
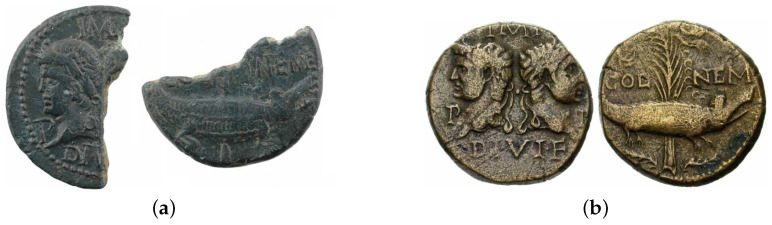
Examples of defective and complete specimens of *Augustus* in our data set. (**a**) An incomplete specimen of RIC 160 of *Augustus*. (**b**) A complete specimen of RIC 160 of *Augustus*.

Although our data set on the whole generally comprises good quality coin samples, a number of images show significantly damaged specimens, that is specimens which are either physically chipped or even cut in half. This is particularly important and noticeable as such specimens are of interest only in case of rare issues and rare issuing authorities, which are for this reason also least abundant in samples, their negative effect on the average performance being amplified by this fact. Examples of such specimens are shown in Figure 11a and Figure 12a, which feature significant semantic information loss as compared with well-preserved samples shown in, respectively, Figure 11b and Figure 12b. The obverse of the coin in Figure 11a is missing the head of Augustus, and the lettering in the top field is barely present. The reverse of the coin shows only the head of the crocodile, with the tree behind it entirely missing.

#### 4.4.2. Worn and Environmentally Affected Coins

**Figure 13:**
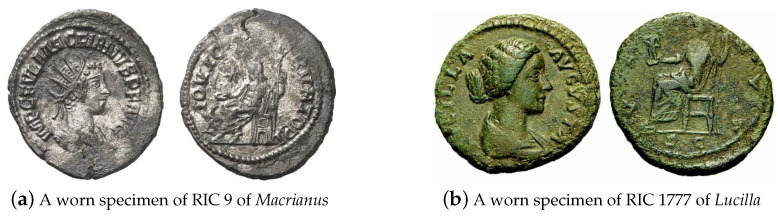
Examples of worn and discolored coins.

As a kind of currency, coins were continuously circulating in ancient times, resulting in surface wear and hence the loss of salient semantic detail crucial for their identification. Exposure to elements, e.g., due to being buried underground, can also effect wear, as well as surface appearance changes in the form of discoloration or patination. All of these factors confound the issue-based matching tasks. At the same time, there are statistical differences in how the coins of different issuers were affected. For example, heavy yet at the time lesser value coins such as sestertii, but which were gradually phased out over time, are more affected by physical wear, see Figure 13b; debased silver coins associated with the period of economic hardship of the Empire in the 3rd century AD are more easily affected by corrosion than good quality silver coins of the early empire, see Figure 13a; and so on.

#### 4.4.3. Data Irregularities

**Figure 14:**
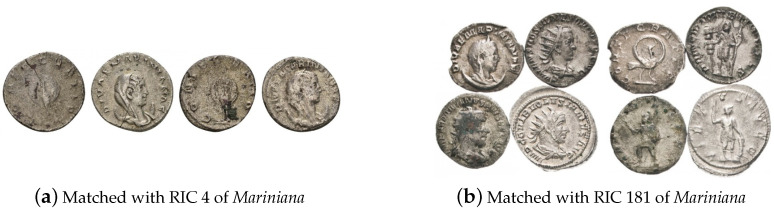
Examples of non-conforming data entries: (**a**) two specimens of Mariniana, also unusually shown reverse first then obverse, and (**b**) four diverse specimens, incorrectly matched as a whole with the issue corresponding to the specimen on the top left.

Recall from Section 4.1 that a normal entry in our data set comprises an image that shows a single coin specimen, its obverse on the left hand side and its reverse on the right hand side, in the natural canonical orientation. However, our examination of problematic text exemplars revealed that a small but not negligible number of the entries in the corpus do not conform with the aforementioned assumption and were not filtered out by our data pre-processing also described in Section 4.1. Examples are shown in Figure 14.

#### 4.4.4. High Similarity between Issues

**Figure 15:**
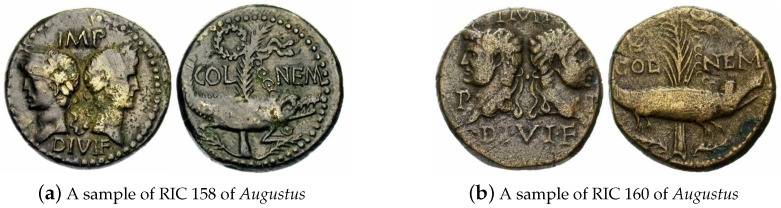
An example of two different issues which are virtually identical in their semantic content.

Lastly, a number of erroneous matches made by our method can be attributed to the inherent difficulty in distinguishing between certain issues that differ in minute detail only. An example is shown in Figure 15, which shows issues RIC 158 and RIC 160 of *Augustus*. These have identical reverses, with the legend COL NEM and the motif showing a crocodile chained to a palm-shoot with long vertical fronds and tip left, and a wreath with long ties above on the left. Their obverses are virtually identical too, with the legend IMP DIVI F and the heads of *Agrippa* (left) and *Augustus* (right) back to back (*Agrippa* wearing a combined rostral crown and laurel wreath, and *Augustus* laureate), the sole difference being the lettering P P in the field of RIC 160. We identified such only subtly different pairs of issues for *Domitia, Saloninus, Macrianus, Fausta, Britannicus, Vabalathus, Julia Paula, Valentinian III,* and *Octavia*.

## 5. Conclusions and Future Work

In this work, our attention was on the problem of image-based ancient coin attribution, which has been at the focus of research on the use of computer vision in ancient numismatics since the nascence of the field. We commenced the article by contextualizing and motivating our key technical contribution, discussing the key limitations of the existing work in the field, both methodological and practical ones. Among the latter, we highlighted the hitherto almost entirely overlooked problem that emerges from the dominant type of approach to ancient coin attribution (namely that in the form of classification), which is the extremely large number of classes (10 s of thousands) for most of which training exemplars are unavailable. This makes the existing algorithms unable to deal with coins of unseen issues, requires a retraining of models when new class exemplars become available, and presents a major class imbalance challenge. Hence, we argued against the classification paradigm and in favor of an alternative. In particular, rather than trying to learn a class specific representation that distinguishes a particular class from all the others, we presented a case for seeking a representation that is overall best at distinguishing classes one from another, thus relinquishing the demand for exemplars of all classes or indeed of any specific class. This led to our adoption of the paradigm of pairwise coin matching by issue, and the specific technical approach in the form of a purpose-crafted Siamese neural network. Furthermore, while adopting deep learning, motivated by its successes in the field and its unchallenged superiority over classical computer vision approaches, we also sought to leverage the advantages that transformers have over the previously employed convolutional neural networks, and in particular their non-local attention mechanisms which ought to be particularly useful in ancient coin analysis by associating semantically but not visually related distal elements of a coin’s design. Finally, we presented a comprehensive and detailed evaluation of the proposed method using a large data corpus of 14,820 images and 7605 issues, and an in-depth analysis of its strengths and weaknesses. Using transfer learning and only a small training set of 542 images of 24 issues, our Double Siamese ViT model was shown to surpass the state of the art by a large margin, achieving an overall accuracy of 81%. Our further investigation of the results showed that the majority of the method’s errors are unrelated to the intrinsic aspects of the algorithm itself, but are rather a consequence of unclean data, which is a problem that can be easily addressed in practice by simple pre-processing and quality checking.

The success of the proposed method and the presented experimental results suggest a number of avenues for further research, which we are currently exploring. Firstly, we expect that an improvement in performance can be effected by training separate Double Siamese ViT models for different kinds of coins: e.g., most coarsely for Roman, Greek, Byzantine, Celtic, etc.; on a finer basis for, e.g., Roman Republican, Roman Imperial preceding the Crisis of the Third Century (which resulted in major changes in both the material and style of coinage), and late Roman Imperial coins; or even for different denominations that exhibit differences both in style and content due to the their different flan sizes and materials used. Secondly, we aim to explore if further informative inference could be made for unknown issues, i.e., issues which are not matched to any gallery ones. The idea here would be to make inferences based on the most similar issues, though not sufficiently similar to produce a match, in a manner conceptually similar to that which has demonstrated success in the context of face recognition, among others [40,41]. 

## Data Availability

The data set used in the present article can be obtained freely for research purposes by contacting the authors.

## Appendix A

**Table A1:** List of possible issuing authorities, including non-conformal entries such as *City Commemoratives*.

Aelia Ariadne	Faustina II	Nero Claudius Drusus
Aelia Flacilla	Flavia Titiana	Nerva
Aelia Verina	Flavius Victor	Nigrinian
Aelius	Florian	Numerian
Aemilian	Gaius and Lucius	Octavia
Agrippa	Galba	Orbiana
Agrippa Postumus	Galeria Valeria	Otacilia Severa
Agrippina I	Galerius	Otho
Agrippina II	Galerius Antoninus	Pacatian
Alexander	Galla Placidia	Paulina
Allectus	Gallienus	Pertinax
Annia Faustina	Gemellus	Pescennius Niger
Annius Verus	Germanicus	Petronius Maximus
Anonymous	Geta	Philip I
Anthemius	Glycerius	Philip II
Antinous	Gordian I	Plautilla
Antonia	Gordian II	Plotina
Antoninus Pius	Gordian III	Poppaea
Aquilia Severa	Gratian	Postumus
Arcadius	Hadrian	Priscus Attalus
Asinius Gallus	Hanniballianus	Probus
Augustus	Helena	Procopius
Aurelian	Herennia Etruscilla	Proculus
Aureolus	Herennius Etruscus	Pulcheria
Avitus	Honoria	Pupienus
Balbinus	Honorius	Quietus
Basiliscus	Hostilian	Quintillus
Bonosus	Johannes	Regalianus
Britannicus	Jotapian	Romulus
Caesonia	Jovian	Romulus Augustus
Caius and Lucius	Jovinus	Sabina
Caligula	Julia	Salonina
Caracalla	Julia Domna	Saloninus
Carausius	Julia Maesa	Sebastianus
Carinus	Julia Mamaea	Sejanus
Carus	Julia Paula	Septimius Severus
City Commemoratives	Julia Soaemias	Severina
Civil Wars	Julia Titi	Severus II
Claudius	Julian I	Severus III
Claudius II (Gothicus)	Julian II	Severus Alexander
Clodius Albinus	Julius Marinus	Statilia Messalina
Clodius Macer	Julius Nepos	Tacitus
Commodus	Laelianus	Tesserae etc.
Constans	Leo I	Tetricus I
Constantine I	Leo II	Tetricus II
Constantine II	Libo	Theodora
Constantine III	Licinia Eudoxia	Theodosius I
Constantius I	Licinius I	Theodosius II
Constantius II	Licinius II	Tiberius
Constantius III	Livia	Titus
Constantius Gallus	Livilla	Trajan
Cornelia Supera	Lucilla	Trajan Decius
Crispina	Lucius Verus	Tranquillina
Crispus	Macrianus	Trebonianus Gallus
Decentius	Macrinus	Uranius Antoninus
Delmatius	Magnentius	Vabalathus
Diadumenian	Magnia Urbica	Valens
Didia Clara	Magnus Maximus	Valentinian I
Didius Julianus	Majorian	Valentinian II
Diocletian	Manlia Scantilla	Valentinian III
Domitia	Marcian	Valeria Messalina
Domitia Lucilla	Marciana	Valerian I
Domitian	Marcus Aurelius	Valerian II
Domitianus	Mariniana	Valerius Valens
Domitilla I	Marius	Varbanov
Domitilla II	Martinian	Varus
Domitius Domitianus	Matidia	Vespasian
Drusus	Maxentius	Vespasian II
Dryantilla	Maximianus	Vetranio
Elagabalus	Maximinus I	Victorinus
Eudocia	Maximinus II	Vindix
Eudoxia	Maximus	Vitellius
Eugenius	Maximus of Spain	Volusian
Fabius Maximus	Nepotian	Zeno
Fausta	Nero	Zenobia
Faustina I	Nero and Drusus Caesars	Zenonis

**Table A2:** Obverse matching performance averaged across each issuing authority.

Issuing Authority	Correct	Total	Rate
Aelius	240	413	58.1%
Aemilian	31	308	10.1%
Agrippa	5	16	31.3%
Agrippina II	5	11	45.5%
Alexander	44	78	56.4%
Allectus	22	82	26.8%
Anthemius	30	45	66.7%
Antoninus Pius	19,832	42,550	46.6%
Aquilia Severa	4	13	30.8%
Arcadius	229	416	55.0%
Augustus	68,606	113,236	60.6%
Aurelian	829	4778	17.4%
Balbinus	24	87	27.6%
Basiliscus	42	57	73.7%
Britannicus	126	215	58.6%
Caligula	585	958	61.1%
Caracalla	6984	17,259	40.5%
Carausius	76	442	17.2%
Carinus	138	565	24.4%
Carus	31	154	20.1%
Claudius	6757	10,063	67.1%
Clodius Albinus	49	159	30.8%
Commodus	4280	9344	45.8%
Constans	1521	4669	32.6%
Constantine I	1340	3327	40.3%
Constantine II	48	106	45.3%
Constantius Gallus	59	156	37.8%
Constantius I	478	1338	35.7%
Constantius II	2360	6581	35.9%
Constantius III	3	15	20.0%
Crispina	87	261	33.3%
Crispus	347	1438	24.1%
Decentius	2	38	5.3%
Delmatius	17	57	29.8%
Diadumenian	23	140	16.4%
Didius Julianus	18	31	58.1%
Diocletian	3226	7163	45.0%
Domitia	6	15	40.0%
Domitia Lucilla	33	106	31.1%
Domitian	7017	12,737	55.1%
Domitianus	3625	6344	57.1%
Domitius Domitianus	19	92	20.7%
Elagabalus	943	3096	30.5%
Eudocia	4	10	40.0%
Eugenius	12	31	38.7%
Fausta	15	48	31.3%
Faustina I	44	233	18.9%
Faustina II	13	45	28.9%
Flavius Victor	6	17	35.3%
Florian	5	79	6.3%
Gaius and Lucius	16	29	55.2%
Galba	1038	2424	42.8%
Galeria Valeria	20	37	54.1%
Galerius	490	1181	41.5%
Gallienus	3239	11,205	28.9%
Germanicus	5448	8804	61.9%
Geta	293	1292	22.7%
Gordian I	2	23	8.7%
Gordian III	565	2095	27.0%
Gratian	32	144	22.2%
Hadrian	25,631	53,702	47.7%
Helena	25	154	16.2%
Herennia Etruscilla	57	111	51.4%
Herennius Etruscus	87	239	36.4%
Honorius	858	1336	64.2%
Hostilian	17	69	24.6%
Johannes	9	16	56.3%
Jovian	11	68	16.2%
Julia	256	501	51.1%
Julia Domna	471	1607	29.3%
Julia Maesa	22	84	26.2%
Julia Mamaea	49	159	30.8%
Julia Paula	11	19	57.9%
Julia Titi	70	123	56.9%
Julian II	128	672	19.0%
Julius Nepos	5	10	50.0%
Leo I	101	155	65.2%
Licinius I	417	1494	27.9%
Licinius II	802	2866	28.0%
Livia	89	126	70.6%
Lucilla	91	261	34.9%
Lucius Verus	1330	2793	47.6%
Macrianus	3	30	10.0%
Macrinus	251	1240	20.2%
Magnentius	216	752	28.7%
Magnia Urbica	30	70	42.9%
Magnus Maximus	106	283	37.5%
Manlia Scantilla	3	11	27.3%
Marcian	107	137	78.1%
Marciana	16	55	29.1%
Marcus Aurelius	57,982	112,714	51.4%
Mariniana	8	25	32.0%
Marius	5	23	21.7%
Maxentius	131	441	29.7%
Maximianus	1209	3677	32.9%
Maximinus I	128	420	30.5%
Maximinus II	52	107	48.6%
Maximus	79	124	63.7%
Nero	7476	14,409	51.9%
Nero Claudius Drusus	57	95	60.0%
Nerva	723	1672	43.2%
Numerian	63	192	32.8%
Octavia	205	502	40.8%
Otacilia Severa	55	264	20.8%
Otho	32	72	44.4%
Pacatian	9	28	32.1%
Pertinax	119	261	45.6%
Pescennius Niger	95	281	33.8%
Philip I	337	1188	28.4%
Philip II	35	143	24.5%
Plautilla	138	307	45.0%
Plotina	6	12	50.0%
Postumus	591	2639	22.4%
Probus	2013	10,033	20.1%
Procopius	34	110	30.9%
Pupienus	42	101	41.6%
Quietus	2	23	8.7%
Quintillus	92	235	39.1%
Romulus	259	570	45.4%
Sabina	75	157	47.8%
Salonina	37	190	19.5%
Saloninus	8	31	25.8%
Septimius Severus	11,676	25,252	46.2%
Severina	17	127	13.4%
Severus Alexander	6477	12,931	50.1%
Severus II	114	213	53.5%
Severus III	6	10	60.0%
Tacitus	131	537	24.4%
Tetricus I	1	21	4.8%
Theodosius I	197	637	30.9%
Theodosius II	865	1442	60.0%
Tiberius	1687	2307	73.1%
Titus	3494	6124	57.1%
Trajan	11,177	25,257	44.3%
Trajan Decius	127	356	35.7%
Tranquillina	22	57	38.6%
Trebonianus Gallus	466	1193	39.1%
Vabalathus	3	22	13.6%
Valens	53	309	17.2%
Valentinian I	28	144	19.4%
Valentinian II	26	139	18.7%
Valentinian III	145	382	38.0%
Valerian I	74	328	22.6%
Vespasian	17,935	31,933	56.2%
Vetranio	65	384	16.9%
Victorinus	68	236	28.8%
Vitellius	3089	5070	60.9%
Volusian	175	675	25.9%
Zeno	107	156	68.6%
	30,2974	613,087	49.4%

**Table A3:** Reverse matching performance averaged across each issuing authority.

Issuing Authority	Correct	Total	Rate
Aelius	293	413	70.9%
Aemilian	133	309	43.0%
Agrippa	12	17	70.6%
Agrippina II	6	10	60.0%
Alexander	39	78	50.0%
Allectus	54	80	67.5%
Anthemius	26	46	56.5%
Antoninus Pius	29,641	42,549	69.7%
Aquilia Severa	10	12	83.3%
Arcadius	310	418	74.2%
Augustus	79,227	113,253	70.0%
Aurelian	2695	4772	56.5%
Balbinus	56	85	65.9%
Basiliscus	32	57	56.1%
Britannicus	75	212	35.4%
Caligula	711	961	74.0%
Caracalla	10,728	17,264	62.1%
Carausius	332	439	75.6%
Carinus	339	565	60.0%
Carus	71	154	46.1%
Claudius	7598	10,066	75.5%
Clodius Albinus	75	160	46.9%
Commodus	6720	9338	72.0%
Constans	2193	4669	47.0%
Constantine I	2271	3328	68.2%
Constantine II	74	105	70.5%
Constantius Gallus	103	153	67.3%
Constantius I	749	1339	55.9%
Constantius II	4472	6577	68.0%
Constantius III	12	15	80.0%
Crispina	168	259	64.9%
Crispus	943	1437	65.6%
Decentius	22	38	57.9%
Delmatius	23	58	39.7%
Diadumenian	89	143	62.2%
Didius Julianus	18	31	58.1%
Diocletian	4950	7168	69.1%
Domitia	10	15	66.7%
Domitia Lucilla	26	107	24.3%
Domitian	8803	12,739	69.1%
Domitianus	4419	6342	69.7%
Domitius Domitianus	52	92	56.5%
Elagabalus	1705	3099	55.0%
Eudocia	4	11	36.4%
Eugenius	16	31	51.6%
Fausta	20	48	41.7%
Faustina I	156	232	67.2%
Faustina II	33	45	73.3%
Flavius Victor	8	17	47.1%
Florian	33	80	41.3%
Gaius and Lucius	19	29	65.5%
Galba	1565	2428	64.5%
Galeria Valeria	12	37	32.4%
Galerius	828	1181	70.1%
Gallienus	7238	11,199	64.6%
Germanicus	4576	8811	51.9%
Geta	688	1289	53.4%
Gordian I	15	22	68.2%
Gordian III	1176	2094	56.2%
Gratian	84	144	58.3%
Hadrian	37,712	53,704	70.2%
Helena	60	156	38.5%
Herennia Etruscilla	82	110	74.5%
Herennius Etruscus	165	236	69.9%
Honorius	942	1337	70.5%
Hostilian	37	68	54.4%
Johannes	8	16	50.0%
Jovian	47	69	68.1%
Julia	370	503	73.6%
Julia Domna	975	1606	60.7%
Julia Maesa	58	84	69.0%
Julia Mamaea	118	159	74.2%
Julia Paula	15	18	83.3%
Julia Titi	102	124	82.3%
Julian II	412	675	61.0%
Julius Nepos	7	10	70.0%
Leo I	102	156	65.4%
Licinius I	916	1494	61.3%
Licinius II	1227	2869	42.8%
Livia	92	126	73.0%
Lucilla	182	263	69.2%
Lucius Verus	2082	2793	74.5%
Macrianus	22	31	71.0%
Macrinus	660	1242	53.1%
Magnentius	539	747	72.2%
Magnia Urbica	36	68	52.9%
Magnus Maximus	176	282	62.4%
Manlia Scantilla	2	11	18.2%
Marcian	105	139	75.5%
Marciana	38	55	69.1%
Marcus Aurelius	79,759	112,717	70.8%
Mariniana	17	25	68.0%
Marius	10	25	40.0%
Maxentius	175	439	39.9%
Maximianus	2509	3675	68.3%
Maximinus I	278	419	66.3%
Maximinus II	62	109	56.9%
Maximus	94	124	75.8%
Nero	10,227	14,412	71.0%
Nero Claudius Drusus	66	94	70.2%
Nerva	1143	1673	68.3%
Numerian	92	192	47.9%
Octavia	280	505	55.4%
Otacilia Severa	185	262	70.6%
Otho	37	73	50.7%
Pacatian	22	28	78.6%
Pertinax	152	260	58.5%
Pescennius Niger	162	278	58.3%
Philip I	785	1186	66.2%
Philip II	97	141	68.8%
Plautilla	163	309	52.8%
Plotina	10	12	83.3%
Postumus	1776	2645	67.1%
Probus	5866	10,030	58.5%
Procopius	60	109	55.0%
Pupienus	68	98	69.4%
Quietus	7	23	30.4%
Quintillus	128	234	54.7%
Romulus	324	567	57.1%
Sabina	94	157	59.9%
Salonina	97	192	50.5%
Saloninus	13	30	43.3%
Septimius Severus	17,822	25,252	70.6%
Severina	80	129	62.0%
Severus Alexander	8472	12,933	65.5%
Severus II	139	211	65.9%
Severus III	7	10	70.0%
Tacitus	325	535	60.7%
Tetricus I	15	22	68.2%
Theodosius I	436	636	68.6%
Theodosius II	731	1444	50.6%
Tiberius	1848	2304	80.2%
Titus	4348	6126	71.0%
Trajan	18,421	25,258	72.9%
Trajan Decius	268	360	74.4%
Tranquillina	46	57	80.7%
Trebonianus Gallus	832	1189	70.0%
Vabalathus	15	22	68.2%
Valens	237	310	76.5%
Valentinian I	99	141	70.2%
Valentinian II	105	139	75.5%
Valentinian III	236	382	61.8%
Valerian I	176	328	53.7%
Vespasian	23,508	31,934	73.6%
Vetranio	199	386	51.6%
Victorinus	165	237	69.6%
Vitellius	3804	5070	75.0%
Volusian	460	677	67.9%
Zeno	89	155	57.4%
	421,686	613,111	68.8%

**Table A4:** Holistic matching performance averaged across each issuing authority.

Issuing Authority	Correct	Total	Rate
Aelius	350	411	85.2%
Aemilian	245	308	79.5%
Agrippa	10	17	58.8%
Agrippina II	7	11	63.6%
Alexander	71	78	91.0%
Allectus	60	80	75.0%
Anthemius	34	46	73.9%
Antoninus Pius	35,169	42,553	82.6%
Aquilia Severa	12	13	92.3%
Arcadius	376	417	90.2%
Augustus	90,666	113,236	80.1%
Aurelian	3666	4773	76.8%
Balbinus	65	86	75.6%
Basiliscus	39	57	68.4%
Britannicus	139	215	64.7%
Caligula	772	962	80.2%
Caracalla	14,570	17,263	84.4%
Carausius	323	439	73.6%
Carinus	454	566	80.2%
Carus	142	155	91.6%
Claudius	8074	10,067	80.2%
Clodius Albinus	119	160	74.4%
Commodus	7681	9343	82.2%
Constans	3283	4667	70.3%
Constantine I	2827	3328	84.9%
Constantine II	89	106	84.0%
Constantius Gallus	124	157	79.0%
Constantius I	1221	1339	91.2%
Constantius II	5616	6579	85.4%
Constantius III	15	15	100.0%
Crispina	221	262	84.4%
Crispus	1120	1438	77.9%
Decentius	32	39	82.1%
Delmatius	48	57	84.2%
Diadumenian	114	143	79.7%
Didius Julianus	25	32	78.1%
Diocletian	5787	7162	80.8%
Domitia	6	15	40.0%
Domitia Lucilla	89	106	84.0%
Domitian	10,417	12,739	81.8%
Domitianus	5269	6344	83.1%
Domitius Domitianus	78	92	84.8%
Elagabalus	2598	3101	83.8%
Eudocia	9	12	75.0%
Eugenius	27	31	87.1%
Fausta	27	47	57.4%
Faustina I	215	232	92.7%
Faustina II	36	45	80.0%
Flavius Victor	14	17	82.4%
Florian	74	80	92.5%
Gaius and Lucius	21	28	75.0%
Galba	1774	2428	73.1%
Galeria Valeria	36	38	94.7%
Galerius	1037	1183	87.7%
Gallienus	9496	11,200	84.8%
Germanicus	6819	8815	77.4%
Geta	1044	1291	80.9%
Gordian I	17	23	73.9%
Gordian III	1739	2097	82.9%
Gratian	123	144	85.4%
Hadrian	44,306	53,709	82.5%
Helena	132	157	84.1%
Herennia Etruscilla	79	109	72.5%
Herennius Etruscus	171	235	72.8%
Honorius	1154	1335	86.4%
Hostilian	54	69	78.3%
Johannes	15	15	100.0%
Jovian	61	69	88.4%
Julia	420	503	83.5%
Julia Domna	1259	1607	78.3%
Julia Maesa	58	83	69.9%
Julia Mamaea	132	160	82.5%
Julia Paula	12	18	66.7%
Julia Titi	115	122	94.3%
Julian II	512	670	76.4%
Julius Nepos	7	10	70.0%
Leo I	126	156	80.8%
Licinius I	1123	1494	75.2%
Licinius II	2102	2868	73.3%
Livia	110	126	87.3%
Lucilla	164	261	62.8%
Lucius Verus	2252	2792	80.7%
Macrianus	18	32	56.3%
Macrinus	1044	1240	84.2%
Magnentius	600	748	80.2%
Magnia Urbica	47	68	69.1%
Magnus Maximus	222	284	78.2%
Manlia Scantilla	10	12	83.3%
Marcian	123	138	89.1%
Marciana	47	56	83.9%
Marcus Aurelius	91,099	112,704	80.8%
Mariniana	13	23	56.5%
Marius	19	26	73.1%
Maxentius	379	439	86.3%
Maximianus	3111	3669	84.8%
Maximinus I	321	422	76.1%
Maximinus II	90	109	82.6%
Maximus	107	123	87.0%
Nero	11,095	14,408	77.0%
Nero Claudius Drusus	74	94	78.7%
Nerva	1343	1674	80.2%
Numerian	160	193	82.9%
Octavia	348	503	69.2%
Otacilia Severa	210	261	80.5%
Otho	64	71	90.1%
Pacatian	23	29	79.3%
Pertinax	202	262	77.1%
Pescennius Niger	222	279	79.6%
Philip I	1013	1188	85.3%
Philip II	124	142	87.3%
Plautilla	234	311	75.2%
Plotina	9	10	90.0%
Postumus	2022	2641	76.6%
Probus	8366	10,039	83.3%
Procopius	84	109	77.1%
Pupienus	72	99	72.7%
Quietus	17	23	73.9%
Quintillus	185	233	79.4%
Romulus	487	565	86.2%
Sabina	113	156	72.4%
Salonina	158	191	82.7%
Saloninus	13	28	46.4%
Septimius Severus	20,869	25,254	82.6%
Severina	99	129	76.7%
Severus Alexander	10,081	12,929	78.0%
Severus II	187	210	89.0%
Severus III	9	10	90.0%
Tacitus	415	535	77.6%
Tetricus I	21	22	95.5%
Theodosius I	548	639	85.8%
Theodosius II	1048	1446	72.5%
Tiberius	1912	2304	83.0%
Titus	4950	6117	80.9%
Trajan	20,731	25,259	82.1%
Trajan Decius	312	356	87.6%
Tranquillina	45	56	80.4%
Trebonianus Gallus	996	1196	83.3%
Vabalathus	15	23	65.2%
Valens	256	312	82.1%
Valentinian I	120	141	85.1%
Valentinian II	113	139	81.3%
Valentinian III	263	381	69.0%
Valerian I	236	328	72.0%
Vespasian	25,734	31,933	80.6%
Vetranio	302	388	77.8%
Victorinus	181	236	76.7%
Vitellius	3945	5077	77.7%
Volusian	580	676	85.8%
Zeno	131	156	84.0%
	496,882	613,110	81.0%
