# Correction: Guo et al. A Siamese Transformer Network for Zero-Shot Ancient Coin Classification. *J. Imaging* 2023, *9*, 107

**DOI:** 10.3390/jimaging10030057

**Published:** 2024-02-26

**Authors:** Zhongliang Guo, Ognjen Arandjelović, David Reid, Yaxiong Lei, Jochen Büttner

**Affiliations:** 1 School of Computer Science, University of St Andrews, Scotland KY16 9AJ, UK; dgr20@st-andrews.ac.uk (D.R.); yl212@st-andrews.ac.uk (Y.L.); 2 Max Planck Institute for the History of Science, Boltzmannstraße 22, 14195 Berlin, Germany; buettner@mpiwg-berlin.mpg.de

## Addition of an Author

Jochen Büttner was not included as an author in the original publication [1]. The corrected Author Contributions statement appears here.

**Author Contributions:** Conceptualization, J.B. (Siamese structure), Z.G. and O.A. (transformer components); methodology, Z.G., O.A. and Y.L.; software, Z.G. and Y.L.; investigation, Z.G. and D.R.; resources, Z.G. and O.A.; data curation, O.A.; writing—original draft preparation, Z.G., O.A., D.R. and Y.L.; writing—review and editing, Z.G. and O.A.; visualization, Z.G.; supervision, O.A.; project administration, O.A.; after initial publication, J.B. has agreed to be added as a co-author. All authors have read and agreed to the published version of the manuscript.

The authors state that the scientific conclusions are unaffected. This correction was approved by the Academic Editor. The original publication has also been updated.
